# Reduction of X-ray-induced DNA damage in normal human cells treated with the PrC-210 radioprotector

**DOI:** 10.1242/bio.035113

**Published:** 2018-08-22

**Authors:** Michael Brand, Matthias Sommer, Frank Jermusek, William E. Fahl, Michael Uder

**Affiliations:** 1 Department of Radiology, Maximiliansplatz 3, University of Erlangen, 91054 Erlangen, Germany; 2 Wisconsin Institutes of Medical Research, University of Wisconsin-Madison, Madison, Wisconsin 53705 USA

**Keywords:** Radioprotector, γ-H2AX immunofluorescence microscopy, Gel electrophoresis, 8-oxo-deoxyguanosine

## Abstract

The aim of our study was to determine the protective efficacy of the PrC-210 aminothiol radioprotector against X-ray-induced DNA damage in normal human cells and to establish dose- and time-effect models for future PrC-210 use in humans. The PrC-210 structure has a branched structure which enables scavenging of reactive oxygen species (ROS) away from DNA. Normal human blood lymphocytes, fibroblasts and naked genomic DNA were exposed to PrC-210 seconds to hours prior to irradiation. Biological (γ-H2AX foci), chemical (8-oxo-deoxyguanosine) and physical (genomic DNA electrophoretic migration) DNA damage endpoints were scored to determine the ability of PrC-210 to suppress radiation-induced DNA damage. X-ray-induced γ-H2AX foci in blood lymphocytes were reduced by 80% after irradiation with 10, 50 and 100 mGy, and DNA double-strand breaks in fibroblasts were reduced by 60% after irradiation with 20 Gy. Additionally, we observed a reduction of 8-oxo-deoxyguanosine (an ROS-mediated, DNA damage marker) in human genomic DNA to background in a PrC-210 dose-dependent manner. PrC-210 also eliminated radiation-induced cell death in colony formation assays after irradiation with 1 Gy. The protective efficacy of PrC-210 in each of these assay systems supports its development as a radioprotector for humans in multiple radiation exposure settings.

## Abstract (precis)

**Summary:** A new strategy is decribed, using a new radioprotector (PrC-210) to significantly reduce radiation-induced DNA damage.

## INTRODUCTION

Human lifetime exposure to ionizing radiation has risen steadily in recent decades, primarily owing to the increased use of diagnostic radiation and computed tomography (CT) in particular. More than 81 million CT scans were done in the United States in 2014 (Des Plaines, 2014), and 3.1 billion radiologic exams were performed worldwide in 2007 (Sodickson et al., 2009; Mettler et al., 2009). The increased use of diagnostic radiation has contributed significantly to radiation exposure in humans and has resulted in studies being conducted concerning the cancer risk associated with radiologic examinations, particularly in children. The quoted estimate for excess cancer mortality from radiation exposure is about 1 death per 2000 CT scans (Berrington de Gonzalez et al., 2009, 2016; Kitahara et al., 2015; Hall, 2002; Linet et al., 2012). Diagnostic radiation risks are particularly pertinent for patients who are genetically predisposed to cancer and therefore useful radiologic procedures are often avoided in their care (Colin and Foray, 2012). A low toxicity, oral radioprotector that could be administered to particular at-risk human populations, in a variety of medical and industrial settings, remains a valid goal.

Irradiation of tissue generates reactive oxygen species (ROS) that cause the large majority of radiation-induced DNA damage (Hall and Giaccia, 2012). Double-strand breaks (DSBs) are regarded as the most serious consequence of radiation exposure that may result in chromosomal translocations and carcinogenesis (Löbrich et al., 2005; Jeggo and Löbrich, 2007; Löbrich and Jeggo, 2007). Chemical modification of DNA by ROS predominantly produces 8-oxo-dG, which is a strongly mutagenic lesion (Gajewski et al., 1990). The addition of phytonutrient antioxidants to cells to induce production of ROS scavenging glutathione, glutathione S-transferase and glutathione peroxidase over hours to days, has shown some radioprotective efficacy in preclinical settings. Amifostine and its active metabolite WR-1065 have shown radioprotective efficacy in preclinical settings (Techapiesancharoenkij et al., 2015; Weiss and Landauer, 2003, 2009; Kuefner et al., 2012; Brand et al., 2015), but these two molecules have severe limiting side effects in humans and they are not orally active, both of which preclude their use as a radioprotector in healthy humans (Rose, 1996).

PrC-210 is the prototype of a new family of small molecule aminothiol radioprotectors which can be administered orally and has no measurable nausea/emesis nor hypotension side effects (Peebles et al., 2012; Copp et al., 2011, 2013; Soref et al., 2012). To evaluate the ability of PrC-210 to suppress X-ray-induced DNA damage in normal human cells we used the H2AX-immunofluorescence microscopy technique to detect γ-H2AX-foci, which is a well-recognized biomarker of radiation-induced DSBs (Rogakou et al., 1998; Rothkamm et al., 2007; Rothkamm and Löbrich, 2003; Rube et al., 2008). This method has been a reliable and sensitive tool for the determination of γ-H2AX-foci induced by radiation (Löbrich et al., 2005; Rothkamm et al., 2007; Rothkamm and Löbrich, 2003; Rube et al., 2008; Brand et al., 2012; Kuefner et al., 2009; Kuefner et al., 2010a,b; Beels et al., 2009). To further evaluate the radioprotective efficacy of PrC-210, we also measured: (i) the chemical level of 8-oxodeoxyguanosine (8-oxo-dG) in X-irradiated human genomic DNA, (ii) colony formation in X-irradiated human fibroblasts and (iii) gel-mobility of DNA from x-irradiated human fibroblasts.

The overall aim of our study was to determine the radioprotective efficacy of PrC-210 in normal human cells and naked DNA to establish dose- and time-effect models for PrC-210 use in humans.

## RESULTS

### Effects of PrC-210 concentration and pre-incubation time on γ-H2AX Foci

**Fig. 1.**
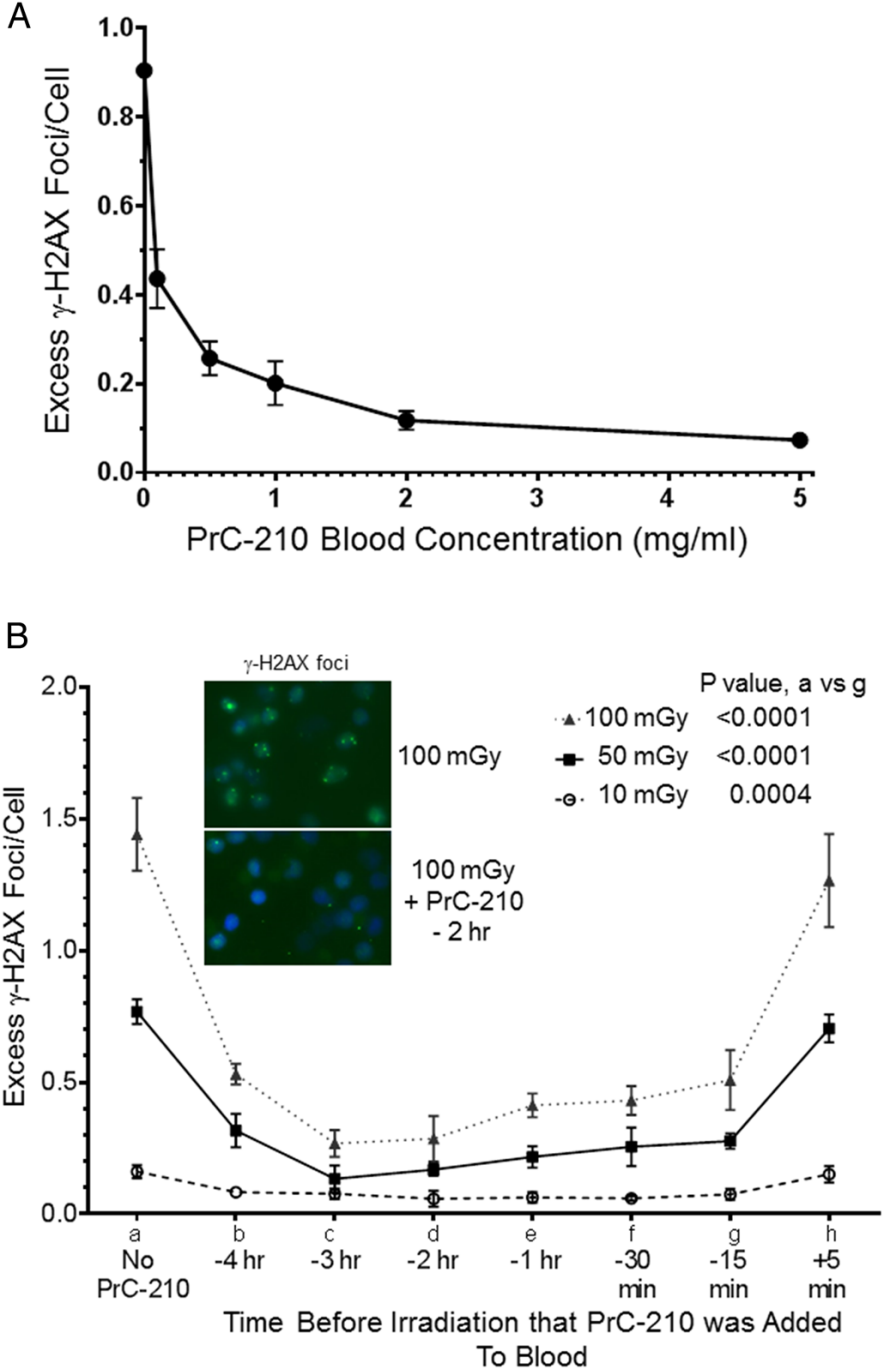
**PrC-210 suppression of γ-H2AX foci in X-irradiated human blood lymphocytes.** Error bars indicates standard deviation. (A) PrC-210 at the indicated concentrations was added to human blood samples 2 h before the 50 mGy irradiation. A significant reduction can be seen at all concentrations with a *P*-value <0.0001. (B) PrC-210 was added to human blood samples at the indicated times before X-irradiation with the indicated doses. *P*<0.0001 except of a versus g at 10 mGy and if PrC-210 was added to blood 5 min after irradiation (*P*-value: a versus h: 0.6304 at 10 mGy, 0.2009 at 50 mGy and 0.3716 at 100 mGy) Insets: Immunostained γ-H2AX foci (green) in human lymphocytes; nuclei were stained with 4,6-diamidino-2-phenylindole.

Pre-incubation of human whole blood with PrC-210 for 2 h before 50 mGy irradiation induced highly significant reductions in γ-H2AX foci at each of the PrC-210 concentrations tested, over a 50-fold concentration range (0.1–5.0 mg/ml; Fig. 1A). At 0.1 mg/ml PrC-210 there was a 52% reduction in foci (*P*=<0.0001) at 1.0 mg/ml PrC-210 a 78% foci reduction (*P*=<0.0001), and at 5.0 mg/ml foci were reduced by 92% compared to the no PrC-210 control (*P*=<0.0001).

A low concentration of PrC-210 (1.0 mg/ml) significantly reduced excess γ-H2AX foci at all pre-incubation times between −4 h to −15 min (Fig. 1B). The reductions in foci when PrC-210 was added 15 min before irradiation were profound: 65% reduction at 100 mGy (*P*<0.0001), 64% reduction at 50 mGy (*P*<0.0001) and 54% reduction at 10 mGy (*P*=0.0004). The largest reduction in X-ray-induced γ-H2AX foci reduction was achieved when PrC-210 was added between −2 h and −3 h (Fig. 1B)

### PrC-210 suppression of X-ray-induced 8-oxo-dG

**Fig. 2.**
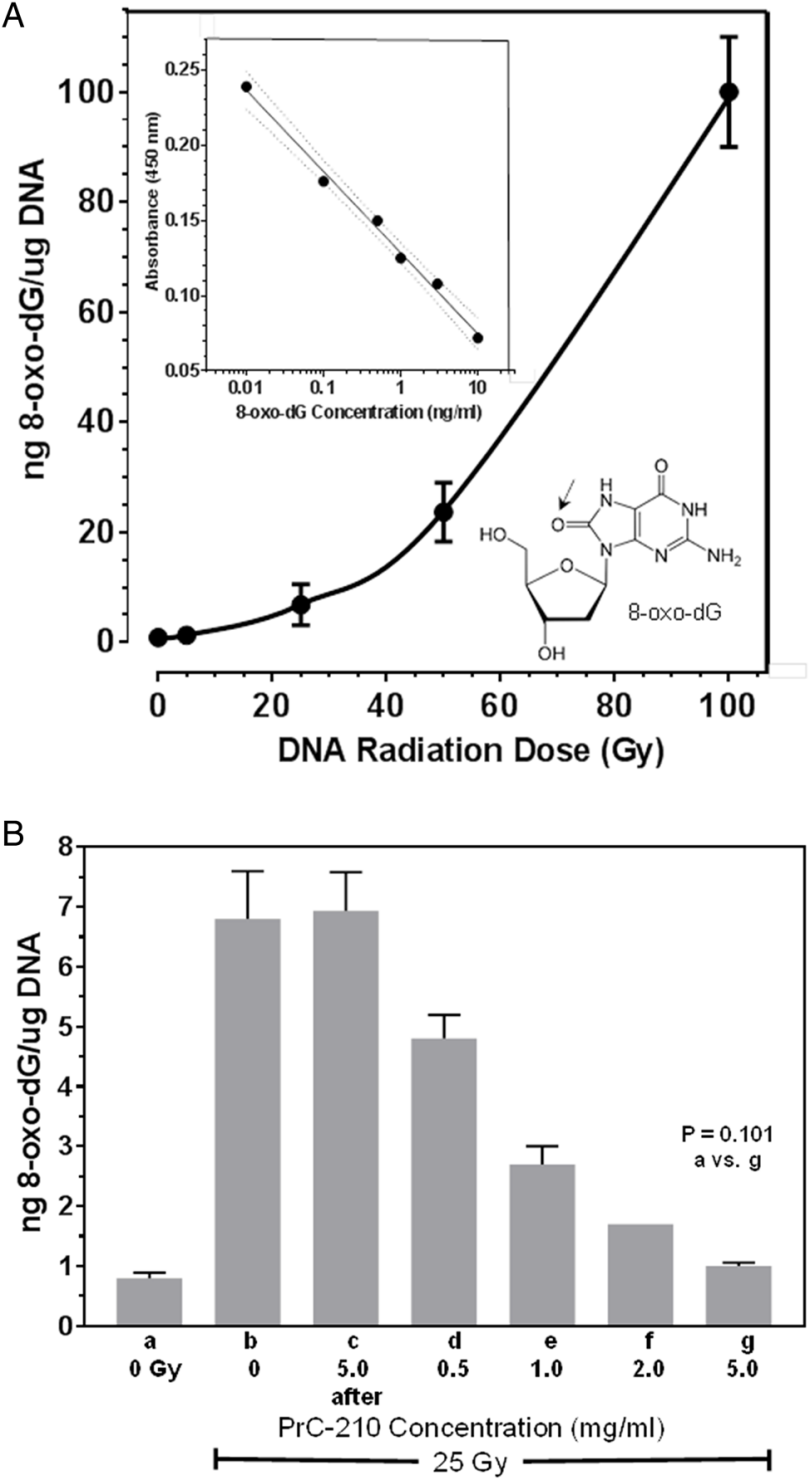
**PrC-210 suppression of 8-oxo-dG formation in X-irradiated human genomic DNA.** Error bars indicates standard deviation. (A) Levels of 8-oxo-dG [see structure of oxygen-modified (arrow) deoxyguanosine] were measured in enzymatic hydrolysates of human genomic DNA that had been irradiated at the indicated X-ray doses. Inset shows ELISA 8-oxo-dG best-fit standard curve with one standard deviation indicated. (B) PrC-210 at the indicated concentrations was added to the genomic DNA incubations 60 min before irradiation of tubes with 25 Gy. Groups contained 3–4 replicates; *P*-value for group a versus g is indicated.

Irradiation of human genomic DNA resulted in the radiation dose-dependent formation of 8-oxo-dG (Fig. 2A), a primary chemical product of ROS attack on DNA (Gajewski et al., 1990). Addition of 0.5-5.0 mg/ml PrC-210 to the DNA 1 h before the 25-Gy irradiation resulted in a PrC-210 dose-dependent suppression of the ROS-oxidized dG base (Fig. 2B). The 8-oxo-dG level in DNA pretreated with 5.0 mg/ml PrC-210 was statistically no different than the 0-mGy, unirradiated control DNA (*P*=0.101).

### Radioprotection of normal human fibroblasts by PrC-210

**Fig. 3.**
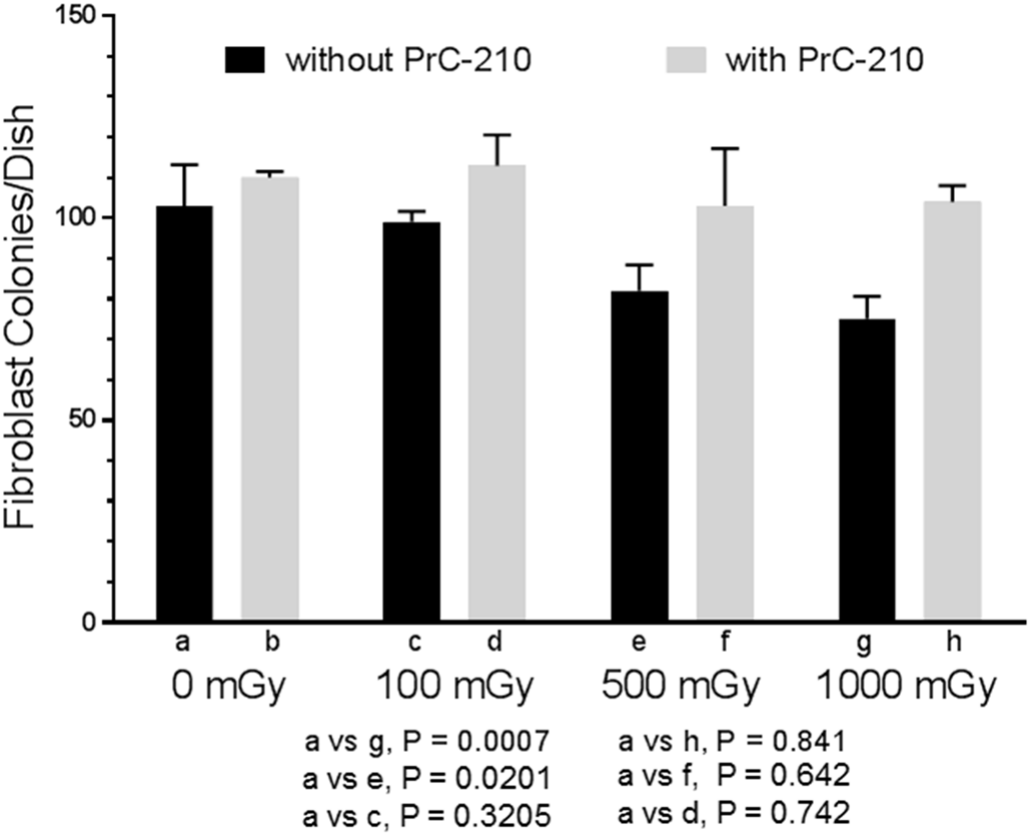
**PrC-210 suppression of X-ray-induced killing of normal human fibroblasts.** Error bars indicates standard deviation. Increasing X-ray doses induced a dose-dependent reduction in fibroblast colony formation. Addition of PrC-210 (1.0 mg/ml) to fibroblast cultures 60 min before irradiation completely suppressed the X-ray-induced cell killing.

**Fig. 4.**
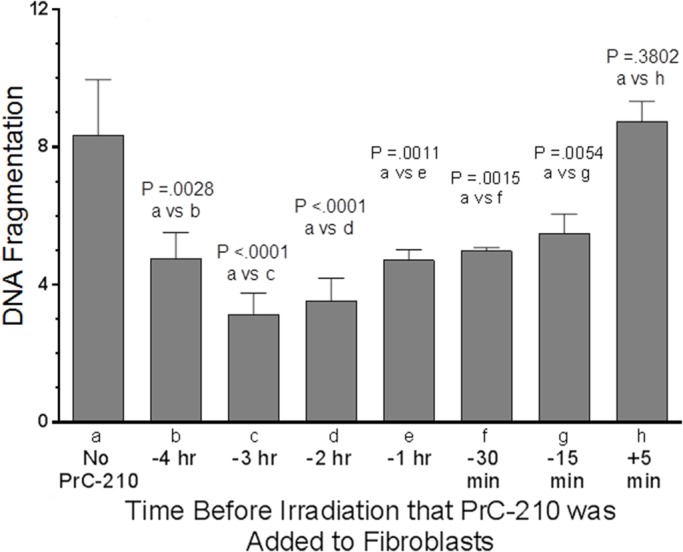
**PrC-210 suppression of DSBs in the genomic DNA of irradiated normal human fibroblasts.** Error bars indicates standard deviation. PrC-210 was added to the cultured fibroblasts at the indicated times before irradiation with 20 Gy. Irradiated cells were digested and their genomic DNA was electrophoresed. *P*-values for comparison of the No PrC-210 group to groups that received PrC-210 at the indicated times of an hour or less before irradiation are indicated. The Y-axis shows the signal intensity of the migrated DNA in relation to the signal intensity of the non-migrated DNA.

Irradiation of normal human fibroblasts in culture produced a significant, radiation dose-dependent reduction in colony formation over the 100–1000 mGy range (Fig. 3). Addition of 1 mg/ml PrC-210 to the tissue culture dishes 1 h before irradiation at all radiation doses resulted in colony formation frequencies that were statistically no different than the 0 mGy controls (Fig. 3). To corroborate the PrC-210-conferred protection of human fibroblasts seen in the colony-forming assay, the same fibroblasts were irradiated at various times after PrC-210 (1 mg/ml) addition to the medium and radiation-induced fragmentation of the fibroblast genomic DNA was scored in a gel mobility assay. A PrC-210 concentration of 1 mg/ml in the culture medium significantly suppressed the number of DSBs at every time that was tested between −15 min and –4 h (Fig. 4). PrC-210 addition at –3 h led to the best damage reduction of 64.5% (*P*<0.0001) but even at −15 min 35% reduction (*P*=0.0054) and at –60 min 44% reduction (*P*=0.0011) were achieved.

## DISCUSSION

Our results demonstrate that the use of PrC-210 leads to a dose-dependent reduction of X-ray-induced γ-H2AX foci in human blood primary lymphocytes, the most sensitive known biological marker of DNA damage, by 50–80% after irradiation with doses between 10–100 mGy. Even after irradiation with 20 Gy we observed a decrease of DNA damage of up to 60% by DNA gel-electrophoresis analysis. In both experimental settings, the largest reduction in DNA damage was seen after a pre-incubation time of 2–3 h. In contrast to our results, some groups noted that a pre-incubation time of 1 h seemed to be most effective both by single antioxidants and by an antioxidants combination (Brand et al., 2015; Kuefner et al., 2012). We can only speculate on this observed effect, maybe PrC-210 needs more time to enter the cell nucleus to intercept free radicals formed near the DNA, but nevertheless a pre-incubation time of 2 h is an acceptable time in patient care. We have chosen 1 mg/ml PrC-210 for the pre-incubation time experiments because we wanted to evaluate a low PrC-210 dose with good effects on DNA damage reduction in view of possible patient studies in the future. Slight but not significant γ-H2AX-foci reduction was observed when adding PrC-210 after irradiation, so the observed effect of PrC-210 on radiation-induced DNA DSBs is caused by a γ-H2AX-foci reduction and not by accelerated γ-H2AX-foci repair. Additionally, X-ray-induced levels of the highly mutagenic oxidized nucleotide 8-oxo-dG, which is formed directly by ROS attack on deoxyguanosine (dG), were reduced to the background level of unirradiated cells by PrC-210 addition.

**Fig. 5.**
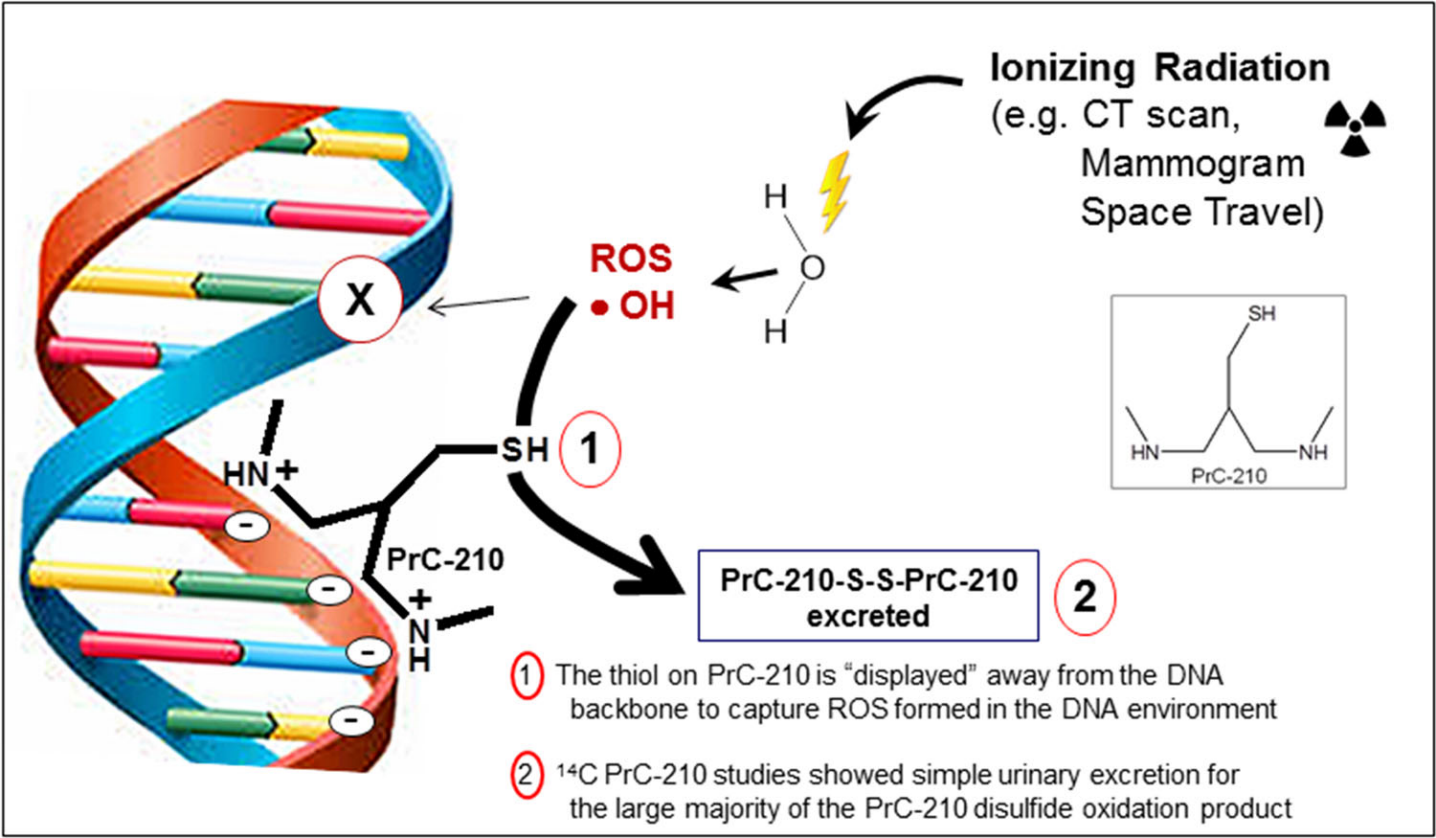
**Schematic showing the primary mechanism by which the PrC-210 aminothiol confers protection against ionizing radiation.** The (+) charged, flexible PrC-210 backbone hovers due to charge above the (−) charged DNA backbone. The ROS scavenging thiol is displaced at three bond lengths away from the DNA to scavenge ROS away from the DNA molecule.

Known antioxidants such as N-acetylcysteine (NAC), vitamin C or vitamin E, which act indirectly by activating Nrf-2 and a battery of Phase II protective molecules, have been shown to reduce X-ray-induced DSBs over a period of hours to days (Kuefner et al., 2012; Brand et al., 2015). PrC-210, however, is a direct-acting ROS scavenger (Peebles et al., 2012; Copp et al., 2013), and when compared to previous antioxidant studies, PrC-210-conferred DSB reduction was significantly higher. For example, in a previous study we observed a DSB reduction of 50% using a cocktail of antioxidants and Phase II enzyme inducers at their saturation doses; this same reduction in DSB was achieved here in 15 min at 20% of the maximum PrC-210 dose tested. We hypothesize that the greater protective efficacy of PrC-210 relates to the basic design of the direct-acting PrC-210 ROS-scavenger, which features a thiol scavenging group that is displayed away from the DNA helix by three bond lengths (Fig. 5).

It is interesting that PrC-210 reduces formation of the mutagenic 8-oxo-dG nucleotide to a level that is statistically not distinguishable from background, but γ-H2AX foci are not reduced to background, even at the highest PrC-210 concentration. This could be explained because the 8-oxo-dG DNA damage marker is formed entirely by the ‘indirect’, ROS-mediated pathway shown in Fig. 5, whereas γ-H2AX immunofluorescence presumably quantifies lesions induced by both the ‘indirect’ ROS-mediated pathway, as well as by the ‘direct’ DNA damage pathway (Hall and Giaccia, 2012), which would presumably be less sensitive to the ROS-scavenging PrC-210 radioprotector.

Cell survival in the colony formation assays was higher after pre-treatment with PrC-210 compared with non-treated cells after irradiation with 1 Gy (Brand et al., 2015). PrC-210 was designed as a direct-acting ROS scavenger that both concentrates and hovers around DNA based upon the positive charge of its backbone; this enables it to act in seconds–minutes (Peebles et al., 2012; Copp et al., 2013). This is unlike most antioxidants and Phase II enzyme inducers, which generally confer protection by inducing glutathione, glutathione-transferase and glutathione-peroxidase synthesis – they generally act in hours–days (Liu et al., 2008). Caution should be exercised in extending the survival information from *in vitro* to *in vivo* settings, but our previous papers have shown similar PrC-210 cytoprotection in animals, for example, complete prevention of Grade 2–3 radiodermatitis when PrC-210 was applied topically or administered by i.p. injection before skin irradiation, and 100% survival of mice from an otherwise 100% lethal dose of whole-body radiation (8.75 Gy) when administered by i.p. injection or oral gavage (Peebles et al., 2012; Copp et al., 2013; Soref et al., 2012).

Quantitation of the γ-H2AX foci biomarker as a means to quantitate X-ray-induced DSBs is a sensitive and proven method that has enabled important assessments of DNA damage encountered at clinical X-ray doses because of its ability to score damage on a cell-by-cell basis. Previous studies have shown that the number of X-ray-induced γ-H2AX foci correlates significantly with the radiation dose and with DNA damage, and the highest γ-H2AX foci levels were measured 5 min after irradiation (Löbrich et al., 2005; Kuefner et al., 2012; Rothkamm and Löbrich, 2003; Rube et al., 2008; Brand et al., 2012; Kuefner et al., 2010a,b; Beels et al., 2009; Rogakou et al., 1998). Due to its unique sensitivity, the γ-H2AX focus assay would be the appropriate assay for initial clinical trials of PrC-210 conferred suppression of X-ray DNA damage.

There are also some limitations in this study. First, the bio-distribution of PrC-210, including mammalian first-pass effects, metabolism and elimination, cannot be well modeled in *in vitro* experiments. The blood PrC-210 concentrations tested here were simply chosen to span the blood concentrations that we achieved in our previous rodent studies (Peebles et al., 2012; Soref et al., 2012). Further, we do not know the tissue and organ concentrations of PrC-210 in the previous animal radioprotection studies. Second, despite the 80+% suppression of X-ray-induced γ-H2AX foci and other DNA damage markers by PrC-210, the PrC-210 effect upon suppression of X-ray-induced cancer is currently unknown. However, tumorigenesis experiments of PrC-210 suppression of X-ray-induced tumors in p53−/− mice are currently underway, and initial data show significant reduction of thymomas in p53−/− mice pretreated with PrC-210 min before 4 Gy irradiation. It should also be noted that there is no increase in tumors in p53−/− mice who receive PrC-210 and 0 Gy radiation, i.e. PrC-210 is not a carcinogen in these mice that are heavily predisposed to cancer induction. This result is consistent with the original observation (Peebles et al., 2012) that PrC-210 is not a bacterial mutagen. The absence of PrC-210 mutagenicity and carcinogenicity is good, but full toxicology testing needs to be done before any clinical trials of the molecule. Third, despite the clear reduction of X-ray-induced DNA damage by PrC-210, its effect upon suppression of cancer risk in irradiated humans remains unknown. Fourth, the radiation doses were different between the experimental settings. For γ-H2AX staining, we chose a radiation dose range that is typically encountered in patientcare (10–100 mGy). γ-H2AX is the most sensitive known biological marker of DNA damage at doses between 10–100 mGy and therefore it is appropriate for this experiment. Unfortunately for the other experiments (DNA gel mobility, colony formation assay and quantitation of the 8-oxo-dG DNA damage marker) we needed to apply higher radiation doses because those methods are not as sensitive as the γ-H2AX immunofluorescence microscopy. Nevertheless, we saw a significant reduction of DNA damage in these assays, even at their higher radiation doses.

The aggregate results of this study show significant suppression of both biological endpoints of DNA damage (representing both indirect and direct DNA damage) as well as chemical endpoints of X-ray-induced DNA damage by achievable blood concentrations of PrC-210 in *in vitro* models, which used both established cell-lines as well as primary blood lymphocytes from ten healthy volunteers. Because of this, we believe that PrC-210 should be pursued as a systemic radioprotector and should be tested in animals and human beings in a clinical setting.

## MATERIALS AND METHODS

This study complies with the Declaration of Helsinki and was performed following local ethic committee approval. Written informed consent was obtained from every volunteer. Exclusion criteria were: X-ray examination within the last 3 days, a history of malignant disease (especially lymphoma or leukemia), radiation therapy or chemotherapy. Blood was obtained from ten healthy volunteers (mean age: 35.5 years; range 28–50 years, five women and five men).

### PrC-210

PrC-210 (MW: 148) has a displayed thiol ROS scavenging group attached to a flexible, alkyl backbone with two charged amines. Synthesis of the PrC-210 aminothiol as an HCl salt is described separately (Copp et al., 2011). To determine what range of human blood PrC-210 concentrations to test here, we estimated that a single PrC-210 intraperitoneal injection at the mouse 0.5×maximum tolerated dose (0.5 MTD) of PrC-210 HCl (0.5 MTD=252 μg/g bw), if fully distributed in the total blood volume of a 21 g mouse, achieves a blood concentration of up to 5.0 mg/ml or 20 mM (i.e. 0.252 mg×21 g=5.29 mg/1.22 ml blood=4.4 mg/ml=19.7 mM). Therefore, for human blood irradiations in this study, 0.1–5.0 mg PrC-210 HCl/ml blood was the tested dose range.

### In vitro blood irradiation experiments

Blood samples (30 ml blood/person for each experiment) were taken using EDTA-containing vials and then incubated under standard conditions (37°C, 5% CO2, 95% air) in 15 ml plastic centrifugation vials (Nunc, Langenselbold, Germany). Work-up of the blood samples for immunostaining began 5 min after irradiation. All experiments were repeated three times for each of the ten volunteers. In each sample, individual baseline levels of γ-H2AX-foci were measured prior to PrC-210 exposure and irradiation. PrC-210 (1.0 mg PrC-210/ml blood) was added to blood samples at 4 h, 3 h, 2 h, 1 h, 30 min and 15 min before irradiating with either 10 mGy, 50 mGy or 100 mGy; PrC-210 was added 5 min after irradiation in one group. Blood samples were also pre-incubated with different PrC-210 concentrations (0.1, 0.5, 1.0, 2.0 or 5.0 mg PrC-210/ml blood) for 2 h and then irradiated with 50 mGy.

### γ-H2AX immunofluorescence microscopy

Staining of γ-H2AX foci in blood lymphocytes is based upon phosphorylation of the histone variant H2AX after formation of DSBs. Irradiated blood samples were layered onto 6 ml of lymphocyte separation medium 1077 (Biochrom, Berlin, Germany) and centrifuged at 1200 ***g*** for 15 min at a temperature of 37°C. The separated, washed and methanol-fixed lymphocytes were stained overnight using a specific γ-H2AX antibody against this phosphorylation (dilution 1:2500) [Anti-H2A.X-Phosphorylated (Ser 139), BioLegend, Uithoorn, The Netherlands]. After incubation with an Alexa Fluor 488-conjugated goat anti-mouse secondary antibody (dilution 1:400) (Invitrogen) the blood lymphocytes were mounted with VECTASHIELD^©^ mounting medium containing 4,6-diamidino-2-phenylindole (Vector Laboratories, Burlingame, USA). Fluorescence analyses were performed with a DM 6000 B microscope (Leica, Wetzlar, Germany) equipped with a 63× and 100× magnification objectives. Cells were counted until 40 γ-H2AX-foci were detected. Every microscope slide was independently counted at least three times by two observers who were blinded to the experiment. Mean values with standard deviations were formed for each blood sample. In order to quantify the γ-H2AX-foci induced by X-ray exposure (so-called ‘excess γ-H2AX foci’) we subtracted the counted γ-H2AX-foci before irradiation (‘background foci’) from the counted γ-H2AX-foci after X-ray exposure. For nuclear staining DAPI (4′,6-diamidino-2-phenylindole) was used.

### Quantitation of the 8-oxo-dG DNA damage marker

Human genomic DNA was purified from Raji cells (#CCL-86) [DNA Isolation Kit for Cells and Tissues (Roche Life Sciences)] that were obtained from ATCC. The cells are a lymphoblastic cell line initially isolated from a Burkitt’s lymphoma (Pulvertraft J.V., 1964). The DNA was irradiated with 5–100 Gy, digested to nucleosides as described (Taghizadeh et al., 2008) and 8-oxodeoxyguanosine (8-oxo-dG) was measured in dilutions of the digests using an ELISA assay (Oxomarker-001 Kit, Health Biomarkers Sweden AB, Stockholm, Sweden) (Taghizadeh et al., 2008; Haghdoost et al., 2005). In some cases, PrC-210 (0.1–5.0 mg/ml) was added to DNA 1 h before 25 Gy irradiation. Irradiated DNA was precipitated and re-dissolved before enzymatic hydrolysis. All enzymes used for DNA digestion to nucleosides were from Sigma-Aldrich. DNA digestion buffers were purged with nitrogen gas to remove oxygen that could raise the 8-oxo-dG background.

### Colony formation assay

For evaluation of cell survival, colony formation assays using human primary skin fibroblasts (TE, wild type, provided by L. Distel, University of Erlangen, Erlangen, Germany) were performed (*n*=10). Cells were plated in petri dishes (1000 cells each) and cultivated in F12 medium supplemented with 12.5% FCS, 1% L-glutamine, 2% nonessential amino acids and penicillin/streptomycin (50 U/ml and 50 μg/ml, respectively). Non-irradiated cells with and without PrC-210 served as controls (0 mGy, no PrC-210; 0 mGy plus 1.0 mg/ml PrC-210). Fibroblasts with and without PrC-210 were irradiated with 100 mGy, 500 mGy or 1000 mGy. After treatment, medium was changed and cells were cultivated for 3 weeks in standard conditions (37°C, 5% CO2, 95% air). Colonies were stained with Methylene Blue and enumerated by two independent observers who were blinded to the experiment.

### DNA gel mobility

Human fibroblasts (TE, wild type, provided by L. Distel) were grown in F12 medium (15% FCS, 1% L-glutamine, 2% nonessential amino acids, and penicillin/streptomycin (50 U/ml and 50 μg/ml) in a humidified incubator at 37°C and 5% CO2 with 95% air (Baierlein et al., 2006). Fibroblasts were pre-incubated with PrC-210 (1.0 mg PrC-210/ml fibroblast suspension) for 4 h, 3 h, 2 h, 1 h, 30 min and 15 min before irradiation; in one group we added PrC-210 5 min after irradiation. All samples were irradiated with 20 Gy. To evaluate induced DSBs the fibroblasts were trypsinized and molded into agarose plugs (0.1 g plug Agarose+10 ml RPMI Medium). After storage and incubation overnight in a solution of proteinase K (1 mg/ml proteinase K, 2% sodium lauryl sulfate) in lysis solution (100 mM EDTA, 50 mM Tris, 50 mM NaCl, pH 8) at 50°C in a shaking water bath, plugs were then dialyzed for 2 h in TBE (0.089 M Tris, 0.089 M Boric acid, 0.0025 M EDTA) and inserted into the wells of a 0.5% agarose gel (0.85 g LE agarose+170 ml 0.5 TBE with SYBR Green (Sigma-Aldrich) at a dilution of 1:10,000. Electrophoresis gels were run for 16 h at 0.85 V/cm. More DSBs mean more fragmented DNA. A fragmented DNA has a better relative mobility in electrophoresis gel (Lumpkin et al., 1985) and therefore also a higher signal intensity after staining. To detect the signal intensity of the stained, migrated, fragmented DNA we used a computer program called Biomas (Erlangen, Germany).

### Statistical analysis

Paired *t*-test and Dunnett's test were used to compare the excess foci pre-treated with PrC-210 to the excess foci without PrC-210 as well as for the 8-oxo-dG and the gel mobility experiments. A *P*-value <0.05 was considered statistically significant. Statistical analysis was performed using the software Prism 7, 2015 (Graph-Pad Software, San Diego, USA).
